# Virtual nursing implementation and 30-day patient readmissions: a multi-site study

**DOI:** 10.1038/s41746-026-02881-2

**Published:** 2026-06-12

**Authors:** Saif Khairat, Zhaoqiang Zhou, Jennifer Morelli, Kevin Joseph Anstrom, Cheryl B Jones

**Affiliations:** 1 Carolina Health Informatics Program, University of North Carolina at Chapel Hill, Chapel Hill, NC USA; 2 School of Nursing, University of North Carolina at Chapel Hill, Chapel Hill, NC USA; 3 The Cecil G. Sheps Center for Health Services Research, University of North Carolina at Chapel Hill, Chapel Hill, NC USA; 4 School of Information and Library Science, University of North Carolina at Chapel Hill, Chapel Hill, NC USA; 5 Gillings School of Global Public Health, University of North Carolina at Chapel Hill, Chapel Hill, NC USA

**Keywords:** Diseases, Health care, Medical research

## Abstract

Current nursing workload and staffing shortages have been associated with adverse patient outcomes, including readmissions. Virtual Nursing (VN) is an emerging care delivery model that shifts selected clinical tasks from bedside nurses to remotely located virtual nurses, but its impact on patient outcomes remains unclear. We conducted a retrospective cohort study across nine hospitals in a major Southeastern U.S. health system, including adult patients discharged from inpatient units during the 12 months before and after VN implementation between 2022 and 2024. VN-assisted discharges were compared with traditional in-person discharges using propensity score matching and staggered difference-in-differences analyses. After matching, 4662 VN-assisted and 4662 in-person discharge encounters were included. Patients discharged through VN had lower 30-day emergency department readmission rates than those receiving traditional discharge care (3.7% vs. 13.3%; *P* < 0.001), despite similar baseline readmission risk scores. VN was associated with a lower readmission risk (risk ratio, 0.28; 95% CI, 0.24–0.33) and absolute risk difference (–0.10; 95% CI, –0.11 to –0.08). Similar reductions were observed in both urban and rural hospitals. These findings suggest that centralized VN-supported discharge may improve discharge processes without worsening short-term patient outcomes.

## Introduction

Current challenges in nurse workload and staffing are associated with worse patient outcomes, such as increased readmission rates^1–3^. With the growing demand for healthcare, hospitals are experiencing nurse shortages and overcrowded emergency departments (ED)^4,5^. These issues result in longer wait times for patients, delayed treatments, and a higher likelihood of readmission due to inadequate initial care^6^. The readmission rates in U.S. hospitals remain high, with some of the most common conditions treated in U.S. hospitals resulting in a readmission rate over 20% within 30 days^7–9^. Frequent readmissions not only strain the healthcare system but also negatively impact patient outcomes and satisfaction^10^.

The quality of nursing care is directly linked to patient outcomes^11,12^. Increased 30-day patient readmission is associated with high mortality rates and increased healthcare costs^13,14^. The quality of patient education during discharge is directly linked with patient readmission rates^15^. When nurses are overworked or understaffed, the quality of admission, discharge, and patient education is compromised, leading to adverse effects on patient outcomes^13,14^.

Job-related stressors, including nursing shortages, can compromise key steps in patient discharge, such as education and medication reconciliation, thereby worsening patient outcomes. Lower nurse staffing levels were significantly associated with higher 30-day readmission rates for heart failure patients^16^. Patients treated in hospitals with better nurse work environments had 12% lower odds of 30-day unplanned readmission compared to those in poorer environments^17^.

In response to these challenges, the implementation of virtual nursing (VN) has emerged as a promising solution within US health systems^18^. Virtual nursing involves the use of technology to provide remote nursing care, allowing nurses to assess, monitor, and communicate with patients virtually^19^. This emerging nursing delivery model has the potential to enhance the quality of clinical tasks by reducing the burden on unit nurses, improving patient flow, and ensuring that patients receive timely care. Additionally, virtual nursing aims to provide patients quicker access to healthcare services, particularly in overcrowded EDs, thereby improving overall patient outcomes and satisfaction^20^.

Within the Job Demands–Resources (JD-R) framework, virtual nursing is considered an organizational resource that mitigates job-related stressors for unit nurses^21–23^. Virtual nurses take on tasks like admission interviews, discharge planning, patient education, and documentation, which helps ease the emotional and workload pressures on bedside staff. This support helps prevent burnout, clarifies roles, and allows unit-based nurses to focus on direct patient care. Virtual nursing serves as a resource that helps balance high job demands, improving well-being, engagement, and performance among frontline nursing staff. As healthcare systems continue to face workforce shortages and spikes in patient acuity, integrating virtual nursing becomes not just a staffing solution but also a strategic resilience mechanism based on organizational theory.

Health systems struggle to examine the return on investment of virtual nursing programs, primarily due to the lack of evaluation metrics and data-driven evidence. Recent studies show that VN improves nursing admission documentation, ED throughput, and nurse and patient satisfaction^24–26^. Despite these improvements, a critical knowledge gap remains regarding the effect of VN on patient outcomes. There is a need for more comprehensive research to evaluate the effectiveness of virtual nursing in various healthcare settings. This gap in the literature underscores the need for further investigation to determine the actual impact of virtual nursing on patient outcomes, including readmission rates.

The central hypothesis was that the implementation of VN in hospitals would improve patient outcomes by reducing ED readmission rates. This is attributed to several factors, including the ability of virtual nursing to deliver effective patient education during discharge, which may improve patient adherence and hence reduce readmission. The objective of this multi-site study was to examine the effect of VN implementation on ED readmission.

## Results

### Study sample

Descriptive statistics of the propensity score-matched sample are shown in Table 1. The matched cohort included 9324 index patient discharge encounters, with 4662 (50%) encounters in each group. Across the matched sample, the average age (SD) was 64.6 (15.6) years, 5272 (57%) were over 65 years, 5001 (54%) were females, 4943 (53%) were White, 9033 (97%) were non-Hispanic, 9191 (99%) reported English as their preferred language, and 9159 (98%) were insured. In terms of clinical characteristics, 5026 (54%) encounters were classified as ESI level 3, and 6788 (73%) were classified as high readmission risk by LACE. With respect to hospital characteristics, 5958 (64%) occurred in urban hospitals, 8021 (86%) in non-teaching hospitals, and 7533 (81%) in medium hospitals. Absolute standardized differences for all measured demographic, socioeconomic, clinical, and hospital characteristics were less than 0.1 after matching, indicating good covariate balance between the two groups^27,28^. Descriptive statistics of the unmatched sample are provided in Supplementary Table 2.

**Table 1:** Descriptive statistics of discharge encounters for propensity score-matched sample

Feature	Overall *N* = 9324	In-person *N* = 4662 (50%)	Virtual nursing *N* = 4662 (50%)	|Standardized difference |
Demographic characteristics				
Age				0.001
Age (years), mean ± SD	64.6 ± 15.6	64.2 ± 14.8	65.1 ± 16.4	
18–34	531 (6%)	268 (6%)	263 (6%)	
35–64	3521 (38%)	1759 (38%)	1762 (38%)	
≥65	5272 (57%)	2635 (57%)	2637 (57%)	
Sex				0.002
Female	5001 (54%)	2498 (54%)	2503 (54%)	
Male	4323 (46%)	2164 (46%)	2159 (46%)	
Race				0.001
White	4943 (53%)	2473 (53%)	2470 (53%)	
Black	3261 (35%)	1630 (35%)	1631 (35%)	
Asian	56 (1%)	28 (1%)	28 (1%)	
American Indian/Alaska Native	783 (8%)	394 (8%)	389 (8%)	
Native Hawaiian or other Pacific Islander	5 (0%)	3 (0%)	2 (0%)	
Other	276 (3%)	134 (3%)	142 (3%)	
Ethnicity				0.011
Hispanic or Latino	248 (3%)	121 (3%)	127 (3%)	
Not Hispanic or Latino	9033 (97%)	4521 (97%)	4512 (97%)	
Other	43 (0%)	20 (0%)	23 (0%)	
Preferred language				0.005
English	9191 (99%)	4597 (99%)	4594 (99%)	
Spanish	85 (1%)	40 (1%)	45 (1%)	
Other	48 (1%)	25 (1%)	23 (0%)	
Socioeconomic characteristics				
Payment type				0.011
Insured	9159 (98%)	4583 (98%)	4576 (98%)	
Self-paid	165 (2%)	79 (2%)	86 (2%)	
Clinical characteristics				
Emergency severity index (ESI)				0.000
Level 1	114 (0%)	60 (1%)	54 (1%)	
Level 2	3975 (43%)	1986 (43%)	1989 (43%)	
Level 3	5026 (54%)	2516 (54%)	2510 (54%)	
Level 4	185 (2%)	90 (1%)	95 (2%)	
Level 5	2 (0%)	1 (0%)	1 (0%)	
Other	22 (0%)	9 (0%)	13 (0%)	
LACE readmission risk				0.004
Low (0–4)	20 (0%)	10 (0%)	10 (0%)	
Medium (5–9)	2516 (27%)	1254 (27%)	1262 (27%)	
High (≥10)	6788 (73%)	3398 (73%)	3390 (73%)	
Hospital characteristics				
Hospital region				0.003
Urban	5958 (64%)	2976 (64%)	2982 (64%)	
Rural	3366 (36%)	1686 (36%)	1680 (36%)	
Hospital type				0.001
Teaching hospitals	1303 (14%)	651 (14%)	652 (14%)	
Non-teaching hospitals	8021 (86%)	4011 (86%)	4010 (86%)	
Hospital scale				0.003
Large (≥500 beds)	1303 (14%)	651 (14%)	652 (14%)	
Medium (100–500 beds)	7533 (81%)	3769 (81%)	3764 (81%)	
Small (<100 beds)	488 (5%)	242 (5%)	246 (5%)	

### 30-day ED readmission

The 30-day ED readmission was significantly lower among encounters discharged with VN than those discharged in-person by the unit nurse (3.7% [95% CI: 3.2%-4.3%] vs. 13.3% [95% CI: 12.4%–14.3%], *P* < .001), Supplementary Table 3. Encounters in the VN group also had a substantially lower risk of 30-day ED readmission than those in the in-person group, with a risk ratio of 0.28 (95% CI: [0.24, 0.33]) and a risk difference of −0.10 (95% CI: [−0.11, −0.08]), indicating both a statistically significant and clinically meaningful reduction in post-discharge ED readmission. The calculated power at a two-sided α level of 0.05 was approximately 1.00, suggesting that the study had adequate power to detect the observed difference.

In the restricted matched sample, the 30-day ED readmission rate remained lower in the VN group than in-person group (4.7% vs. 7.3%), corresponding to a risk ratio of 0.64 (95% CI: [0.45, 0.91]) and risk difference of −2.63 percentage points (95% CI: [−4.68, −0.58]) (*P* = 0.012), Supplementary Table 4. These findings were consistent in direction with the primary encounter-level analysis and suggest that the primary results were not solely driven by repeated eligible discharges from the same patient.

### Subgroup analysis

**Fig. 1:**
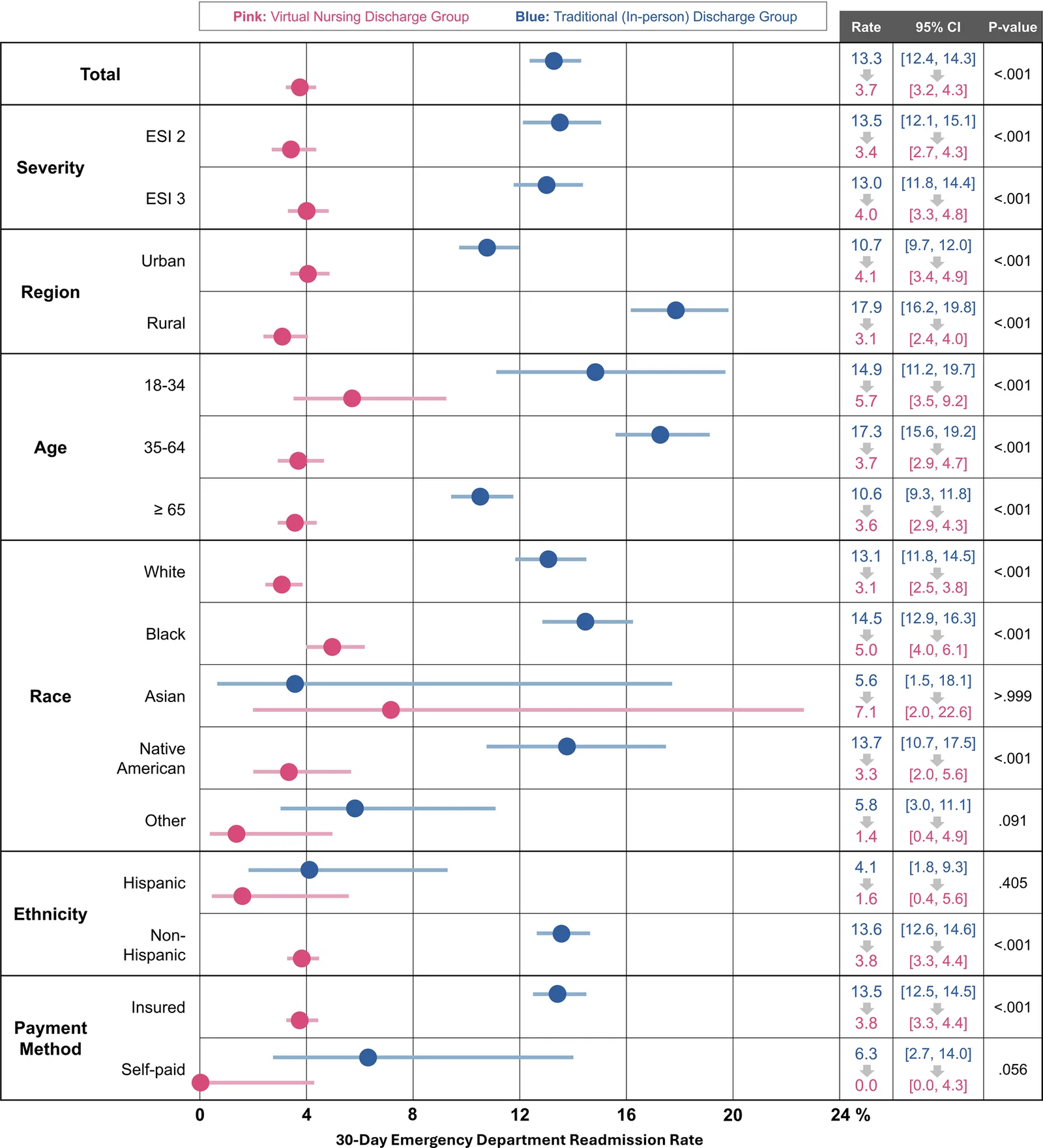
Thirty-day emergency department readmission rates with 95% confidence intervals by subgroup for virtual nursing and in-person discharge encounters. 30-day emergency department readmission rates are shown for patient encounters discharged through the virtual nursing model and through traditional in-person discharge. Pink circles and horizontal lines indicate the readmission rate and 95% confidence interval for the virtual nursing discharge group. Blue circles and horizontal lines indicate the readmission rate and 95% confidence interval for the traditional in-person discharge group. The right-hand table reports the readmission rate, 95% confidence interval, and P-value for each subgroup analysis. Subgroups include overall encounters and strata defined by Emergency Severity Index level, hospital region, age group, race, ethnicity, and payment method. Emergency Severity Index levels 2 and 3 are shown because these encounters accounted for more than 90% of the analytic sample.

ESI levels 2 and 3 were the most common severity categories, accounting for approximately 97% of the matched sample. Within these subgroups, 30-day ED readmission was consistently lower among VN discharge encounters than in-person discharge encounters. For ESI level 2, the 30-day ED readmission rate was 3.4% (95% CI: 2.7%–4.3%) in the VN group versus 13.5% (95% CI: 12.1%–15.1%) in the in-person group (*P* < 0.001). For ESI level 3, the corresponding rates were 4.0% (95% CI: 3.3%–4.8%) versus 13.0% (95% CI: 11.8%–14.4%) (*P* < 0.001), and for ESI level 4, 2.1% (95% CI: 0.6%–7.4%) versus 13.3% (95% CI: 7.8%–21.9%) (*P* < 0.001). ESI levels 1 and 5 were infrequent, and subgroup-specific estimates for those categories were considered imprecise because of small sample size, Fig. 1 and Supplementary Table 5. Interaction testing did not indicate statistically significant heterogeneity by ESI subgroup.

Subgroup analysis by hospital region also showed lower 30-day ED readmission rates in the VN group than in-person group. In urban hospitals, the 30-day ED readmission rate was 4.1% (95% CI: 3.4%–4.9%) for VN discharge versus 10.7% (95% CI: 9.7%–12.0%) for in-person discharge (*P* < 0.001). In rural hospitals, the corresponding rates were 3.1% (95% CI: 2.4%–4.0%) versus 17.9% (95% CI: 16.2%–19.8%) (*P* < 0.001), Fig. 1 and Supplementary Table 6. Interaction testing indicated significant heterogeneity by hospital region (*P* < 0.001).

Across most demographic, socioeconomic, and clinical subgroups, 30-day ED readmission rates were lower in the VN group than in-person group. Estimates were less precise in smaller subgroups, including Asian patients, patients categorized as other race, Hispanic patients, and self-paid patients, Fig. 1.

### Association analysis

In the multivariable logistic regression model (AUC = 0.64), VN discharge was associated with significantly lower odds of 30-day ED readmission compared with in-person discharge (OR 0.24 [95% CI: 0.20–0.29], *P* < 0.001), Table 2. Male patients had lower odds compared to female patients (OR 0.42 [95% CI: 0.36–0.50], *P* < 0.001). Patients aged 65 years or older had lower odds compared to those aged 18–34 years (OR 0.49 [95% CI: 0.35–0.67], *P* < 0.001). American Native patients had lower odds compared to White patients (OR 0.63 [95% CI: 0.47–0.85], *P* = 0.002), and Hispanic patients had lower odds compared to non-Hispanic patients (OR 0.37 [95% CI: 0.15–0.90], *P* = 0.028). Patients discharged from urban hospitals had lower odds compared to rural hospitals (OR 0.68 p95% CI: 0.57–0.80), *P* < 0.001). Patients discharged from teaching hospitals had lower odds compared to non-teaching hospitals (OR 0.10 [95% CI: 0.03–0.33], *P* = 0.001). Medium-sized hospitals showed lower odds compared to large hospitals (OR 0.19 [95% CI: 0.06–0.60], *P* = 0.004), and small hospitals also had lower odds compared to large hospitals (OR 0.05 [95% CI: 0.02–0.18], *P* < 0.001). Conversely, higher LACE readmission risk was associated with significantly greater odds of 30-day ED readmission (OR 1.67 [95% CI: 1.38–2.02], *P* < 0.001). In a sensitivity analysis replacing the categorized LACE readmission risk variable with its component measures, the association between VN discharge and lower 30-day ED readmission remained materially similar (OR 0.19 [95% CI: 0.15–0.25], *P* < 0.001), supporting the robustness of the adjusted findings to an alternative specification of baseline readmission risk, Supplementary Table 7.

**Table 2:** Multivariable logistic regression results for factors associated with 30-day ED readmission

Variable	OR (95% CI)	*P*-value
VN usage		
VN vs. In-person discharge	0.24 (0.20–0.29)	<0.001***
Demographic characteristics		
Sex		
Male vs. female	0.42 (0.36–0.50)	<0.001***
Age, year		
18–34	1 [Reference]	-
35–64	0.84 (0.62–1.16)	0.295
≥ 65	0.49 (0.35–0.67)	<0.001***
Race		
White	1 [Reference]	-
Black	1.05 (0.89–1.24)	0.555
American Indian/Alaska Native	0.63 (0.47–0.85)	0.002**
Other	0.63 (0.30–1.33)	0.226
Ethnicity		
Hispanic vs. non-Hispanic	0.37 (0.15–0.90)	0.028*
Preferred language		
English vs. other languages	1.87 (0.64–5.46)	0.251
Clinical characteristics		
Emergency severity index (ESI)		
Level 2	1 [Reference]	-
Level 3	0.94 (0.80–1.09)	0.414
Level 4	0.78 (0.44–1.38)	0.390
LACE readmission risk		
High (≥10) vs. Medium (5–9)	1.67 (1.38–2.02)	<0.001***
Hospital characteristics		
Hospital region		
Urban vs Rural	0.68 (0.57–0.80)	<0.001***
Hospital type		
Teaching hospital vs. non-teaching hospital	0.10 (0.03–0.33)	0.001**
Hospital scale		
Large (≥500 beds)	1 [Reference]	-
Medium (100–500 beds)	0.19 (0.06–0.60)	0.004**
Small (<100 beds)	0.05 (0.02–0.18)	<0.001***

* The factors with insufficient cases, including Asian, Pacific Islander, self-paid, ESI level 1 and 5, low LACE score, were not included in the model.

### Difference-in-difference analysis

All seven treatment cohorts exhibited statistically significant group-time ATT estimates. The aggregated ATT across cohorts and post-implementation periods was –0.015 (95% CI: [–0.019, –0.011], *P* < 0.001), indicating an average absolute reduction of approximately 1.5 percentage points in the 30-day ED readmission rate following VN implementation at the hospital level, Supplementary Table 8.

**Fig. 2:**
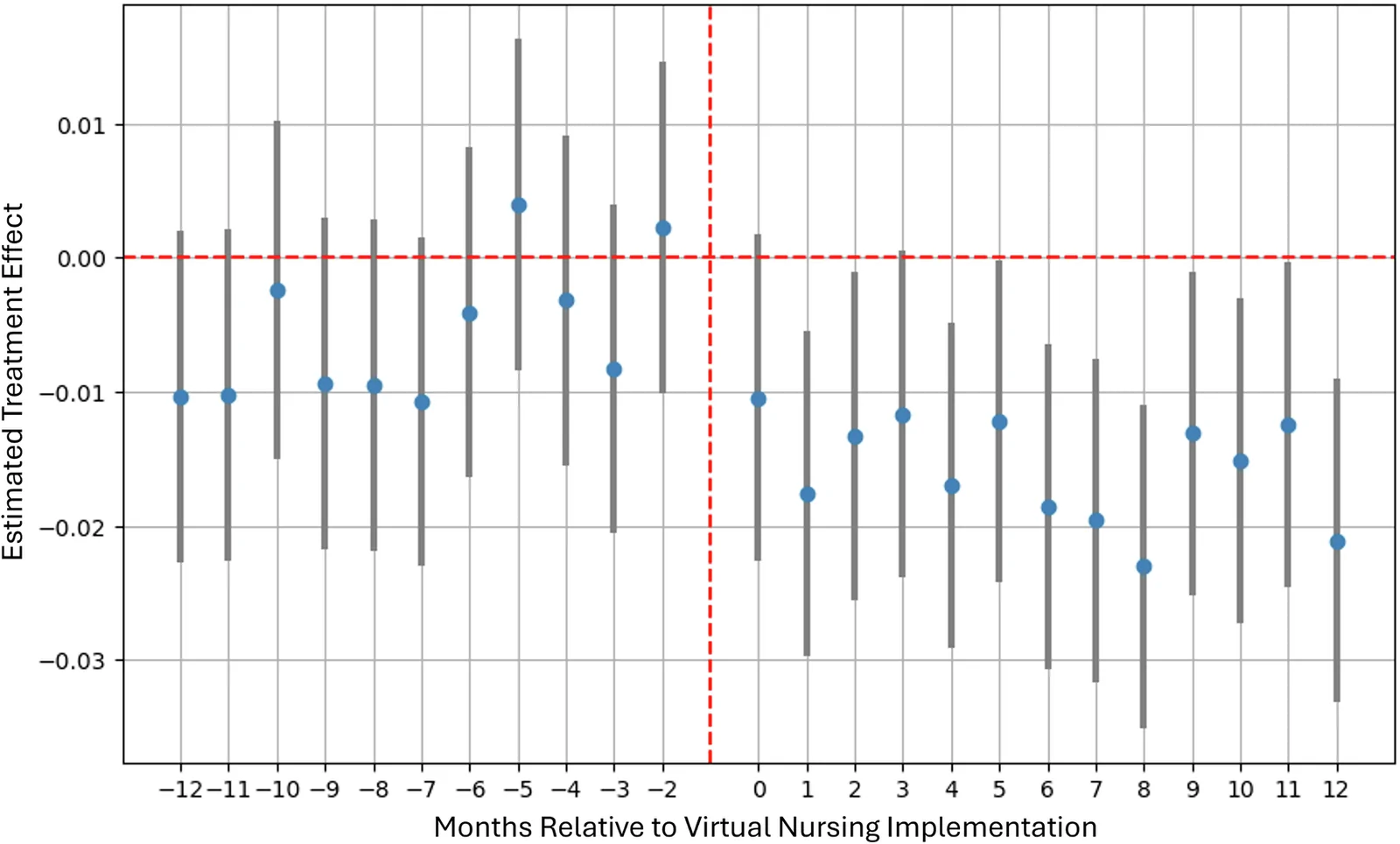
Event study of average treatment effects with 95% confidence intervals for 30-day emergency department readmission by months relative to virtual nursing implementation. Event-study estimates show average treatment effects in 30-day emergency department readmissions before and after virtual nursing implementation. Blue circles indicate estimated treatment effects for each month relative to implementation, and gray vertical lines indicate 95% confidence intervals. The red horizontal dashed line indicates no difference in the outcome, and the red vertical dashed line indicates the timing of virtual nursing implementation.

Event study supported the parallel trends assumption. Estimated ATTs during the pre-implementation period (relative months -12 to 0) were not statistically significant in most months, with the exception of month +3, Fig. 2 and Supplementary Table 9.

The magnitude of the aggregated DiD estimate was smaller than that observed in the propensity score-matched post-implementation comparison, as expected, because the two analyses addressed different estimates. The matched comparison reflects the association between VN discharge and 30-day ED readmission among encounters that actually received VN during the post-implementation period, whereas the DiD analysis estimates the average hospital-level effect of VN rollout across the full eligible population over time, including many encounters that did not receive VN during the early rollout phase. Accordingly, the DiD estimate represents a system-level implementation effect rather than a recipient-level contrast.

## Discussion

In this multi-site retrospective study across nine hospitals, VN discharge was associated with lower 30-day ED readmission compared with in-person discharge. Approximately 1 in 27 VN patients was readmitted to the ED within 30 days, compared to 1 in 8 in-person patients. The association was observed in the propensity score-matched post-implementation comparison, remained directionally consistent in a sensitivity analysis restricted to the first eligible index inpatient discharge per patient, and was supported by multiple complementary analytic approaches. This analysis provides initial, real-world evidence that virtual nursing can streamline discharge without evidence of harm on short-term acute-care return.

The reduction in 30-day ED readmissions points to potential strengths in the VN care delivery model. Discharge is a complex, vulnerable transition often compromised by unit noise, interruptions, and nurse workload. The structured, dedicated, and uninterrupted interaction facilitated by a virtual nurse, focused on medication reconciliation, teach-back, and care plan review, may enhance the quality and consistency of discharge education. This improved process likely contributes to better patient understanding and self-management after leaving the hospital. Furthermore, the consistency of benefit across urban and rural hospitals is notable. The effect appeared even more pronounced in rural settings, where access to specialized support can be limited, suggesting VN could be a strategic tool for improving care equity and quality in geographically dispersed health systems.

Several complementary mechanisms may plausibly explain the observed association. First, VN encounters may increase fidelity to core discharge processes, including medication review, follow-up scheduling, and patient education, because the virtual nurse’s role is dedicated to discharge and is less likely to be interrupted by competing bedside tasks. Second, remote delivery may introduce greater standardization across units and hospitals using shared scripts, checklists, and EHR templates, reducing between-clinician variability that can undermine discharge quality. Third, the VN model may improve team communication by offloading administrative and educational tasks from unit nurses, allowing bedside staff to focus on last-mile coordination (e.g., ensuring prescriptions are ready). Finally, the encounter itself may improve patient engagement. The structure of a dedicated, one-on-one video interaction may reduce ambient distraction, signal to patients that the discharge conversation warrants full attention, and lower the threshold for patients and caregivers to ask questions.

At the same time, alternative explanations should be considered. Because VN discharge was not randomized and was offered to patients who were alert and responsive, the VN group may have differed in unmeasured ways (e.g., caregiver availability) that are not fully captured by ESI, LACE, or other observed covariates. Operational factors may also contribute: VN encounters may have been preferentially used during periods of higher unit workload, at particular discharge hours, or on units with stronger discharge cultures, which could correlate with lower ED return rates independent of the modality itself. Although propensity score matching, regression adjustment, and the staggered DiD analysis reduce confounding from measured factors, residual confounding remains possible; therefore, these results should be interpreted as evidence of association and as a rationale for prospective evaluation of mechanisms (e.g., direct measures of discharge comprehension and medication discrepancies) in future studies.

Findings from our study on the implementation of virtual nursing in hospitals have significant implications for the future of healthcare delivery. VN has demonstrated its potential to enhance efficiency and support bedside nurses, with evidence that patient outcomes are at least maintained. The findings also align with and extend the Job Demands-Resources (JD-R) framework applied to healthcare^22^. By offloading the comprehensive discharge process to a dedicated virtual nurse, the model acts as a tangible organizational resource. It directly reduces the task burden and cognitive load on bedside nurses during a critical workflow, potentially mitigating a key job demand. This resource reallocation may allow unit-based nurses to focus on direct, hands-on patient care and other complex tasks, theoretically improving both nurse well-being and unit performance. The high utilization of VN among older adults also counters assumptions about technological barriers, indicating that careful design and facilitation can make such models accessible across age groups.

Our study builds upon a growing literature examining virtual nursing models, which have primarily focused on intermediate outcomes such as documentation completeness, nurse satisfaction, and emergency department throughput^20,24,26^. Previous work has posited the potential for VN to improve care quality but has been limited by a lack of data on definitive patient outcomes^19^. This study addresses that critical gap by providing quantitative, multi-site evidence linking VN to patient-potential enhancements in care quality and system efficiency. We extend the existing evidence base by demonstrating that the theorized benefits of VN for workflow and satisfaction may translate into measurable improvements in patient outcomes.

Several limitations must be considered. First, the observational design means that selection into VN discharge was not randomized and depended on patient eligibility and clinician/patient choice. Although we adjusted for a wide array of observed demographic, clinical, and hospital factors using PSM and regression, the possibility of residual confounding by unmeasured variables (e.g., patient health literacy, social support, or specific clinician attitudes) persists. Second, the intervention was limited to the discharge encounter and did not include structured post-discharge support. In addition, because the intended discharge workflow was substantively similar between VN and in-person discharge and the principal differences were the mode of delivery and staffing configuration, we could not isolate the independent effect of the virtual modality itself from that of a dedicated discharge staffing model. Relatedly, discharge-process mediators such as communication quality, patient comprehension, and the quality of medication reconciliation were not directly measured, so mechanistic interpretations should remain cautious. Third, the primary outcome captured readmissions to participating EDs only, so ED use outside the health system could be missed. Fourth, the study was conducted within a single health system in the Southeastern United States, which may limit generalizability. Fifth, the VN service operated from 7 am to 7 pm, so effects might differ for late-evening discharges. Sixth, early rollout meant relatively low adoption overall, which likely attenuated system-level estimates. Seventh, eligibility for VN discharge, average time spent per discharge, and reasons for non-coverage were not systematically captured in structured analytics fields, limiting our ability to fully characterize intervention uptake and workflow differences. Eighth, the study period was limited to 12 months post-implementation, preventing assessment of the long-term sustainability of the observed effect. Finally, although subgroup analyses suggested differences between rural and urban hospitals, this finding should be interpreted cautiously as the intervention was limited to the discharge encounter and did not include structured regional subgroup differences in baseline risk and other contextual factors.

Moving forward, it is essential to conduct further research, including randomized controlled trials and cost-effectiveness studies, to validate these findings and explore the long-term impact of VN on patient outcomes and hospital outcomes. Additionally, integrating VN as a job resource rather than a substitute for in-person nursing staff appears to promote further improvements in standard practice by enabling a more efficient, effective, and patient-centered healthcare system.

In conclusion, the implementation of a virtual nursing model for hospital discharge can reduce short-term ED readmissions at the bedside and, as adoption grows, contribute to modest system-level gains. For hospital leaders, the practical implication is to scale virtual discharge thoughtfully to embed a standardized education and teach-back workflow, track adoption share, and monitor revisit rates so that operational benefits do not come at the expense of patient outcomes.

## Methods

### Study design

This retrospective cohort study employed a propensity score-matched (PSM) comparison and a staggered difference-in-differences (DiD) design to investigate the effect of implementing VN in hospitals across a major Southeastern healthcare system in the United States. Participating hospitals varied in size and included both rural and urban settings, enabling assessment of the VN discharge model across diverse hospital environments and patient populations. VN was implemented in these hospitals between 2022 and 2024 through dedicated VN centers. This study adhered to the Strengthening the Reporting of Observational Studies in Epidemiology (STROBE) guidelines for cohort studies, and the checklist is provided in Supplementary Table 1^29^. The study was approved by the University of North Carolina at Chapel Hill Office of Human Research Ethics (IRB number: 23-2580), with a waiver of informed consent as it used health record data retrospectively and posed minimal risk to participants. All study procedures followed institutional requirements for protecting patient privacy and confidentiality.

### Intervention

The intervention was a centralized VN discharge service delivered through telehealth by remotely located registered nurses. The intended discharge workflow was substantively similar to in-person discharge. In both modalities, discharge encounters were intended to include review of the discharge summary, medication instructions, follow-up recommendations, and patient education using the same institutional discharge processes and electronic health records (EHR)-based documentation framework. The principal difference was the mode of delivery and staffing configuration, rather than a distinct discharge curriculum or post-discharge support program.

VN services were delivered by a centralized team of experienced registered nurses located in an off-site virtual nursing center. All recruited virtual nurses were previously bedside nurses within the health system to ensure awareness of institutional processes and clinical workflow. Virtual nurses connected with patients via a secure, HIPAA-compliant, EHR-integrated video platform accessible on bedside tablets in participating hospital units. VN encounters were documented within the institutional EHR using the same documentation framework as standard care.

The inpatient unit nurse offers the VN discharge assessment option to patients being discharged from participating inpatient units. To qualify for a VN encounter, the patient had to be alert and responsive. For patients who accepted VN discharge, the bedside nurse prepared the tablet and notified the virtual nurse through the EHR that the patient is ready. The virtual nurse then conducted the discharge encounter by video, including review of discharge instructions, medication guidance, follow-up recommendations, and patient education. These encounters emphasized confirmation of patient understanding of the care plan through teach-back. At the end of the session, the virtual nurse communicated completion of the discharge encounter and any next steps to the bedside nurse through the EHR.

The VN service operated 7 days per week from 7 am to 7 pm, with staffing adjusted to anticipated discharge volumes.

### Participants and data

The study used deidentified encounter-level data from nine hospitals within a major Southeastern U.S. health system that shared a common institutional EHR. Data collection covered 12 months before and 12 months after VN implementation for each participating hospital to ensure adequate hospital-specific pre- and post-implementation observation windows. The unit of analysis was the inpatient discharge encounter.

Eligible encounters were limited to adults aged 18 years or older who presented to the ED of a participating hospital and were subsequently admitted to an inpatient unit. There were no exclusions based on sex, race, or ethnicity. Demographic variables included age, sex, race, ethnicity, and preferred language; socioeconomic variables included primary payer types; clinical variables included Emergency Severity Index (ESI) and LACE readmission-risk measures; and hospital variables included region, teaching status, and bed size. ESI is a five-level ED triage measure to categorize patients, with level 1 indicating the highest urgency and level 5 the lowest. LACE is a clinical readmission-risk index for predicting 30-day readmission risk based on the aggregate score of length of stay, acuity of admission, comorbidities burden, and prior ED use within the last 6 months, providing a clinically meaningful summary of factors strongly associated with 30-day readmission^30^. There was no missing data.

### Measurements and outcomes

The primary outcome was 30-day readmission to a participating ED, defined as any readmission to the ED of a participating hospital within 30 days after discharge from the index inpatient encounter. Outcome ascertainment was limited to participating hospitals within the shared institutional EHR; therefore, ED readmissions to nonparticipating hospitals were not captured. The outcome was analyzed as a binary event at the discharge level, such that an index discharge was classified as having a 30-day ED readmission if at least one qualifying readmission occurred during follow-up. Multiple ED readmissions within the same 30-day follow-up period were not counted separately. The 30-day ED readmission rate is a comprehensive measure of the quality of hospital services, discharge management, and coordination of the healthcare system^31^.

### Statistical analysis

The primary analysis compared 30-day ED readmission between discharge encounters that received VN assessment and those that received in-person assessment during the 12-month post-implementation period. Because VN implementation dates varied across hospitals (with seven distinct implementation dates across nine hospitals), we first extracted the hospital-specific 12-month post-implementation data for each participating hospital and then pooled these data to form the post-implementation analytic cohort.

To reduce confounding by observed characteristics, we estimated a propensity score for each encounter^32^, defined as the conditional probability of receiving VN discharge given baseline covariates:

$${\rm{e}}\left({{\rm{X}}}_{{\rm{i}}}\right)=\Pr ({{\rm{Z}}}_{{\rm{i}}}=1|{{\rm{X}}}_{{\rm{i}}})$$

where ${{\rm{Z}}}_{{\rm{i}}}$ indicates receipt of VN discharge for encounter ${\rm{i}}$, and ${{\rm{X}}}_{{\rm{i}}}$ denotes the vector of demographic, socioeconomic, clinical, and hospital characteristics. Propensity scores were estimated using logistic regression:

$${\rm{logit}}({\rm{e}}({{\rm{X}}}_{{\rm{i}}}))={{\rm{\alpha }}}_{0}+{{\rm{X}}}_{{\rm{i}}}^{{\rm{T}}}{\rm{\alpha }}.$$

We then performed 1:1 nearest-neighbor matching without replacement to construct a matched in-person comparison group for the VN group. Covariate balance after matching was assessed using absolute standardized differences with values less than 0.1 considered indicative of acceptable balance^33^.

In the matched post-implementation sample, we compared 30-day ED readmission between VN and in-person discharge encounters using multiple complementary effect measures. For each group, the event rate was estimated as:

$${\hat{{\rm{p}}}}_{{\rm{g}}}=\frac{1}{{{\rm{n}}}_{{\rm{g}}}}\mathop{\sum }\limits_{{\rm{i}}:{{\rm{Z}}}_{{\rm{i}}}={\rm{g}}}{{\rm{Y}}}_{{\rm{i}}}$$

where ${{\rm{Y}}}_{{\rm{i}}}$ is an indicator for 30-day ED readmission and ${\rm{g}}\in \left\{0,1\right\}$ denotes VN or in-person discharge. We summarized group differences using the risk ratio

$${\rm{RR}}=\frac{{\hat{{\rm{p}}}}_{{\rm{VN}}}}{{\hat{{\rm{p}}}}_{{\rm{IP}}}}$$

and the risk difference

$${\rm{RD}}={\hat{{\rm{p}}}}_{{\rm{VN}}}-{\hat{{\rm{p}}}}_{{\rm{IP}}}$$

with corresponding 95% confidence intervals (CIs) and additionally chi-square tests.

We also conducted subgroup analyses to evaluate whether associations differed by patient severity and hospital context. Subgroups were defined by ESI, hospital region (urban vs rural), and demographic and socioeconomic characteristics. Within each subgroup, we estimated 30-day ED readmission rates with 95% CIs for VN and in-person discharge. Effect heterogeneity was assessed by including an interaction term between discharge modality and subgroup variable in a regression model and evaluating the statistical significance of the interaction term

To further investigate underlying factors contributing to variations in 30-day ED readmission, we fit a multivariable logistic regression model of the form:

$${\rm{logit}}\left\{{\rm{P}}\left({{\rm{Y}}}_{{\rm{i}}}=1|{{\rm{Z}}}_{{\rm{i}}},{{\rm{X}}}_{{\rm{i}}}\right)\right\}={{\rm{\beta }}}_{0}+{{\rm{\beta }}}_{1}{{\rm{Z}}}_{{\rm{i}}}+{{\rm{X}}}_{{\rm{i}}}^{{\rm{T}}}{\rm{\beta }}$$

where ${{\rm{Y}}}_{{\rm{i}}}$ indicates 30-day ED readmission, ${{\rm{Z}}}_{{\rm{i}}}$ indicates VN discharge, and ${{\rm{X}}}_{{\rm{i}}}$ indicates demographic, clinical, and hospital characteristics. Adjusted odds ratios (ORs) and 95% CIs were reported.

To estimate the hospital-level effect of VN rollout over time, we adopted a staggered DiD analysis using the group-time average treatment effect on the treated (ATT) estimator proposed by Callaway and Sant’Anna^34^. Let ${{\rm{G}}}_{{\rm{i}}}$ denote the treatment cohort defined by implementation timing and ${\rm{t}}$ index calendar time. The group-time ATT is defined as:

$${\rm{ATT}}\left({\rm{g}},{\rm{t}}\right)={\rm{E}}\left[{{\rm{Y}}}_{{\rm{t}}}(1)-{{\rm{Y}}}_{{\rm{t}}}(0)\right|{{\rm{G}}}_{{\rm{i}}}={\rm{g}}].$$

This analysis used the full eligible population without PSM and included the 12-month pre- and 12-month post-implementation periods for each hospital. We reported both group-time specific ATT estimates and the aggregated ATT across treated hospital groups and treatment periods. Standard errors were clustered at the group level to account for within-group correlation over time. To assess the plausibility of the parallel trend assumption, we examined event-study estimates by relative month and tested whether pre-treatment lead coefficients were statistically distinguishable from zero.

The propensity score-matched post-implementation comparison and the staggered DiD analysis were intended to address complementary questions. The matched comparison estimated the association between VN discharge and 30-day ED readmission among encounters that actually received VN discharge during the post-implementation period, whereas the staggered DiD analysis estimated the average hospital-level effect of VN rollout across the full eligible population over time.

### Sensitivity analysis

Because patients could contribute more than one eligible index inpatient discharge during the study period, we conducted a sensitivity analysis restricted to the first eligible index inpatient discharge per patient. This analysis was intended to assess whether repeated observations from the same patient influenced the primary encounter-level findings. In the restricted post-implementation cohort, propensity scores were re-estimated using the same baseline covariates as in the primary analysis, nearest-neighbor PSM was repeated, and the association between discharge modality and 30-day ED readmission was re-estimated using the same effect measures as in the primary analysis.

To improve the interpretability of the adjusted models, we conducted an additional sensitivity analysis in which the categorized LACE readmission-risk variable was replaced by its component measures in the adjusted regression model. Specifically, variables corresponding to length of stay, acuity of admission, comorbidity burden, and prior ED utilization were represented as dichotomized indicators (high vs low) based on the sample median. Since the acuity-of-admission component conceptually overlapped with the ESI-based severity adjustment used in the primary model, ESI was omitted from the component-based sensitivity specification.

### Statistical infrastructure and software

All analyses were performed in Python 3.13.5 using libraries NumPy, Pandas, SciPy, Scikit-learn, and Statsmodels. A *p*-value less than 0.05 was considered statistically significant.

## Supplementary information

Supplementary information

## Data Availability

The data used by this study were derived from electronic health records. It is not publicly available because they contain protected health information and are subject to institutional review board approval, HIPAA regulations, data use agreements, and health system privacy policies. The authors are not authorized to share the underlying patient-level data outside the approved study team.

## Code availability

No custom software or algorithm was developed for this study. Statistical analyses were conducted using standard statistical software. Analysis code may be made available from the corresponding author upon reasonable request, subject to institutional policies and restrictions related to the underlying electronic health record data.
