# Supplementary material for: Virtual nursing implementation and 30-day patient readmissions: a multi-site study
Source: NPJ Digit Med. 2026 Jun 12;9:734. doi: 10.1038/s41746-026-02881-2 (PMC13616903; doi:10.1038/s41746-026-02881-2)
Supplement: Supplementary file 1 [file 41746_2026_2881_MOESM1_ESM.pdf]

Supplements

Supplementary Table 1. STROBE checklist for cohort studies

	Item No	Recommendation	Reported in the Article
**Title and abstract**	1	(*a*) Indicate the study's design with a commonly used term in the title or the abstract	Title, Abstract
		(*b*) Provide in the abstract an informative and balanced summary of what was done and what was found	Abstract
**Introduction**			
Background/rationale	2	Explain the scientific background and rationale for the investigation being reported	Introduction
Objectives	3	State specific objectives, including any prespecified hypotheses	Introduction
**Methods**			
Study design	4	Present key elements of study design early in the paper	Methods
Setting	5	Describe the setting, locations, and relevant dates, including periods of recruitment, exposure, follow-up, and data collection	Methods, Intervention, Participants and Data
Participants	6	(*a*) Give the eligibility criteria, and the sources and methods of selection of participants	Methods, Participants and Data
Variables	7	Clearly define all outcomes, exposures, predictors, potential confounders, and effect modifiers. Give diagnostic criteria, if applicable	Methods, Participants and Data, Measurements and Outcomes, Statistical Analysis
Data sources/ measurement	8*	For each variable of interest, give sources of data and details of methods of assessment (measurement). Describe comparability of assessment methods if there is more than one group	Methods, Participants and Data, Measurements and Outcomes, Statistical Analysis
Bias	9	Describe any efforts to address potential sources of bias	Methods, Statistical Analysis
Study size	10	Explain how the study size was arrived at	Methods, Participants

			and Data, Statistical Analysis
Quantitative variables	11	Explain how quantitative variables were handled in the analyses. If applicable, describe which groupings were chosen and why	Methods, Statistical Analysis
Statistical methods	12	(*a*) Describe all statistical methods, including those used to control for confounding	Methods, Statistical Analysis
		(*b*) Describe any methods used to examine subgroups and interactions	Methods, Statistical Analysis
		(*c*) Explain how missing data were addressed	Methods, Participants and Data
		(*d*) If applicable, describe analytical methods taking account of sampling strategy	Methods, Statistical Analysis
		(*e*) Describe any sensitivity analyses	N/A
**Results**			
Participants	13*	(a) Report numbers of individuals at each stage of study—eg numbers potentially eligible, examined for eligibility, confirmed eligible, included in the study, completing follow-up, and analysed	Results
		(b) Give reasons for non-participation at each stage	Results
		(c) Consider use of a flow diagram	N/A
Descriptive data	14*	(a) Give characteristics of study participants (eg demographic, clinical, social) and information on exposures and potential confounders	Results, Table 1
		(b) Indicate number of participants with missing data for each variable of interest	N/A
Outcome data	15*	Report numbers of outcome events or summary measures	Results, Difference-in-Difference Analysis
Main results	16	(*a*) Give unadjusted estimates and, if applicable, confounder-adjusted estimates and their precision (eg, 95% confidence interval). Make clear which confounders were adjusted for and why they were included	Results, Readmission, Subgroup Analysis
		(*b*) Report category boundaries when continuous variables were categorized	N/A
		(*c*) If relevant, consider translating estimates of relative risk into absolute risk for a meaningful time period	Results, Readmission, Subgroup Analysis, Difference-in-Difference

			Analysis
Other analyses	17	Report other analyses done—eg analyses of subgroups and interactions, and sensitivity analyses	Results, Subgroup Analysis, Association Analysis, Difference-in-Difference Analysis
**Discussion**			
Key results	18	Summarise key results with reference to study objectives	Discussion
Limitations	19	Discuss limitations of the study, taking into account sources of potential bias or imprecision. Discuss both direction and magnitude of any potential bias	Discussion
Interpretation	20	Give a cautious overall interpretation of results considering objectives, limitations, multiplicity of analyses, results from similar studies, and other relevant evidence	Discussion
Generalisability	21	Discuss the generalisability (external validity) of the study results	Discussion
**Other information**			
Funding	22	Give the source of funding and the role of the funders for the present study and, if applicable, for the original study on which the present article is based	Funding Statement

Supplementary Table 2. Descriptive statistics of patient encounters without PSM processing

Feature	Overall N = 66,827	In-Person N = 62,159 (93%)	Virtual Nursing N = 4,668 (7%)	|Standardized Difference|
**Demographic characteristics**				
Age				.066
Age (years), Mean ± SD	63.9 ± 18.1	63.9 ± 18.2	65.1 ± 16.4	
18-34	5,607 (8%)	5,343 (9%)	264 (6%)	
35-64	24,626 (37%)	22,861 (37%)	1,765 (38%)	
≥ 65	36,594 (55%)	33,955 (55%)	2,639 (57%)	
Sex				.021
Female	34,573 (52%)	32,065 (52%)	2,508 (54%)	
Male	32,252 (48%)	30,092 (48%)	2,160 (46%)	
Race				.057
White	38,446 (58%)	35,972 (58%)	2,474 (53%)	
Black	20,989 (31%)	19,356 (31%)	1,633 (35%)	
Asian	591 (1%)	563 (1%)	28 (1%)	
American Indian/Alaska Native	2,984 (4%)	2,595 (4%)	389 (8%)	
Native Hawaiian or other Pacific Islander	55 (0%)	53 (0%)	2 (0%)	
Other	3,762 (6%)	3,620 (6%)	142 (3%)	
Ethnicity				.023
Hispanic or Latino	2,992 (4%)	2,865 (5%)	127 (3%)	
Not Hispanic or Latino	63,089 (94%)	58,571 (94%)	4,518 (97%)	

Other	746 (1%)	723 (1%)	23 (0%)	
Preferred language				.022
English	64,320 (96%)	59,720 (96%)	4,600 (99%)	
Spanish	1,861 (3%)	1,816 (3%)	45 (1%)	
Other	646 (1%)	623 (1%)	23 (0%)	
**Socioeconomic characteristics**				
Payment type				.021
Insured	64,280 (96%)	59,698 (96%)	4,582 (98%)	
Self-paid	2,547 (4%)	2,461 (4%)	86 (2%)	
**Clinical characteristics**				
Emergency Severity Index (ESI)				.026
Level 1	2,656 (4%)	2,602 (1%)	54 (1%)	
Level 2	27,442 (41%)	25,451 (41%)	1,989 (43%)	
Level 3	35,037 (52%)	32,523 (52%)	2,510 (54%)	
Level 4	1,495 (2%)	1,400 (2%)	95 (2%)	
Level 5	35 (0%)	34 (0%)	1 (0%)	
Other	162 (0%)	149 (0%)	13 (0%)	
LACE readmission risk				.012
Low (0-4)	211 (0%)	201 (0%)	10 (0%)	
Medium (5-9)	17,240 (26%)	15,987 (26%)	1,262 (27%)	
High (≥ 10)	49,056 (74%)	45,666 (74%)	3,390 (73%)	
**Hospital characteristics**				
Hospital region				.011
Urban	35,887 (54%)	32,903 (53%)	2,984 (64%)	
Rural	30,940 (46%)	29,256 (47%)	1,684 (36%)	
Hospital type				.246
Teaching hospitals	24,617 (37%)	23,965 (39%)	652 (14%)	
Non-teaching hospitals	42,210 (63%)	38,194 (61%)	4,016 (86%)	
Hospital scale				.275
Large (≥ 500 beds)	24,617 (37%)	23,965 (39%)	652 (14%)	
Medium (100-500 beds)	35,495 (53%)	31,727 (51%)	3,768 (81%)	
Small (< 100 beds)	6,715 (10%)	6,467 (10%)	248 (5%)	

Supplementary Table 3. 30-day ED readmission statistics for in-person and VN patient encounters

Variable		In-person N = 4,662	Virtual Nursing N = 4,662	Risk Ratio with 95% CI	Risk Difference with 95% CI	P-value
30-day ED readmission				0.28 (0.24, 0.33)	-0.10 (-0.11, -0.08)	<.001***
	Count	620	174			
	Rate	13.3%	3.7%			
	95% CI	12.4%-14.3%	3.2%-4.3%			

Supplementary Table 4. Sensitivity analysis restricted to the first eligible index inpatient discharge per patient

Variable		In-person N = 1,026	Virtual Nursing N = 1,026	Risk Ratio with 95% CI	Risk Difference with 95% CI	P-value
30-day ED readmission				0.64 (0.45, 0.91)	-2.63 (-4.68, -0.58)	.012*
	Count	75	48			
	Rate	7.3%	4.7%			

Supplementary Table 5. 30-day ED readmission statistics for in-person and VN patient encounters by ESI

Variable		In-person N = 4,662	Virtual Nursing N = 4,662	Risk Ratio with 95% CI	Risk Difference with 95% CI	P-value	Interaction P-value
**ESI level 1**		N = 60	N = 54				
30-day ED readmission				0.28 (0.08, 0.93)	-0.14 (-0.26, -0.03))	.252	-
	Count	12	3				
	Rate	20.0%	5.6%				
	95% CI	11.8%-31.8%	1.9%-15.1%				
**ESI level 2**		N =1,986	N = 1,989				
30-day ED readmission				0.25 (0.20, 0.33)	-0.10 (-0.12, -0.08)	<.001***	.958
	Count	268	68				
	Rate	13.5%	3.4%				
	95% CI	12.1%-15.1%	2.7%-4.3%				
**ESI level 3**		N = 2,516	N = 2,510				
30-day ED readmission				0.31 (0.25, 0.38)	-0.09 (-0.11, -0.08)	<.001***	.813
	Count	328	101				
	Rate	13.0%	4.0%				
	95% CI	11.8%-14.4%	3.3%-4.8%				
**ESI level 4**		N = 90	N = 95				
30-day ED readmission				0.16 (0.04, 0.69)	-0.11 (-0.19, -0.04)	<.001***	.614
	Count	12	2				
	Rate	13.3%	2.1%				
	95% CI	7.8%-21.9%	0.6%-7.4%				
**ESI level 5**		N = 1	N = 1				
30-day ED readmission				-	-	-	-
	Count	0	0				
	Rate	0.0%	0.0%				
	95% CI	-	-				

Supplementary Table 6. 30-day ED readmission statistics for in-person and VN patient encounters by region

Variable		In-person N = 4,662	Virtual Nursing N = 4,662	Risk Ratio with 95% CI	Risk Difference with 95% CI	P-value	Interaction P-value
**Urban**		N = 2,976	N = 2,982				
30-day ED readmission				0.38 (0.31, 0.46)	-0.07 (-0.08, -0.05)	<.001***	-
	Count	319	122				
	Rate	10.7%	4.1%				
	95% CI	9.7%-12.0%	3.4%-4.9%				
**Rural**		N = 1,686	N = 1,680				
30-day ED readmission				0.17 (0.13, 0.23)	-0.15 (-0.17, -0.13)	<.001***	<.001***
	Count	301	52				
	Rate	17.9%	3.1%				
	95% CI	16.2%-19.8%	2.4%-4.0%				

Supplementary Table 7. Sensitivity analysis replacing the composite LACE readmission-risk variable with its component measures

Variable	OR (95% CI)	P-value
**VN usage**		
VN vs In-person discharge	0.19 (0.15-0.25)	<.001***
**Demographic characteristics**		
Sex		
Male vs Female	0.43 (0.36-0.53)	<.001***
Age, year		
18-34	1 [Reference]	-
35-64	0.86 (0.58-1.30)	.479
≥ 65	0.39 (0.26-0.59)	<.001***
Race		
White	1 [Reference]	-
Black	1.05 (0.86-1.29)	.616
American Indian/Alaska Native	0.62 (0.46-0.84)	.002**
Other	0.88 (0.35-2.26)	.796
Ethnicity		
Hispanic vs non-Hispanic	0.48 (0.15-1.53)	.216
Preferred language		
English vs Other Languages	0.94 (0.26-3.42)	.923
**Clinical characteristics**		
LACE component (high vs low)		
Length of stay	0.63 (0.50-0.79)	< .001***
Acuity of admission	1.20 (0.99-1.44)	.058
Comorbidities	0.88 (0.68-1.15)	.354
ED visits in the last 6 months	1.11 (1.03-1.19)	< .001***

Hospital characteristics		
Hospital region		
Urban vs Rural	0.46 (0.37-0.58)	< .001***
Hospital type		
Teaching hospital vs non-teaching hospital	0.16 (0.05-0.50)	.002**
Hospital scale		
Large (≥ 500 beds)	1 [Reference]	-
Medium (100-500 beds)	0.28 (0.09-0.87)	.028*
Small (< 100 beds)	0.08 (0.02-0.27)	<.001***

Supplementary Table 8. ATT estimates from difference-in-difference analysis of VN implementation effects on 30-day ED readmission by hospital treatment group based on implementation time

Treatment Group	ATT	SE	95% CI	P-value
**30-day ED readmission**				
Aggregated Average	-0.015	0.002	[-0.019, -0.011]	<.001***
2022-08	-0.025	0.003	[-0.030, -0.020]	<.001***
2023-01	-0.025	0.002	[-0.029, -0.021]	<.001***
2023-06	-0.013	0.002	[-0.017, -0.010]	<.001***
2023-07	-0.011	0.002	[-0.015, -0.008]	<.001***
2023-08	-0.009	0.002	[-0.013, -0.006]	<.001***
2023-12	-0.010	0.002	[-0.014, -0.007]	<.001***
2024-01	-0.010	0.002	[-0.014, -0.006]	<.001***

Supplementary Table 9. ATT estimates from difference-in-difference analysis of VN implementation effects on 30-day ED readmission by relative month

Relative Month	ATT	SE	95% CI	P-value
**30-day ED readmission**				
-12	-0.010	0.006	[-0.023, 0.002]	.099
-11	-0.010	0.006	[-0.023, 0.002]	.105
-10	-0.002	0.006	[-0.015, 0.010]	.705
-9	-0.009	0.006	[-0.022, 0.003]	.134
-8	-0.009	0.006	[-0.021, 0.003]	.132
-7	-0.011	0.006	[-0.023, 0.002]	.087
-6	-0.004	0.006	[-0.016, 0.008]	.515
-5	0.004	0.006	[-0.008, 0.016]	.529
-4	-0.003	0.006	[-0.015, 0.009]	.609
-3	-0.008	0.006	[-0.021, 0.004]	.186
-2	0.002	0.006	[-0.010, 0.015]	.723
-1	[Reference]	-	-	-
0	-0.010	0.006	[-0.023, 0.002]	.092
1	-0.018	0.006	[-0.030, -0.005]	.004**

2	-0.013	0.006	[-0.025, -0.001]	.033*
3	-0.012	0.006	[-0.024, 0.000]	.060
4	-0.017	0.006	[-0.029, -0.005]	.006**
5	-0.012	0.006	[-0.024, -0.000]	.046*
6	-0.019	0.006	[-0.030, -0.006]	.003**
7	-0.020	0.006	[-0.032, -0.008]	.001**
8	-0.023	0.006	[-0.035, -0.011]	<.001***
9	-0.013	0.006	[-0.025, -0.001]	.033*
10	-0.015	0.006	[-0.027, -0.003]	.015*
11	-0.012	0.006	[-0.025, -0.000]	.043*
12	-0.021	0.006	[-0.033, -0.009]	<.001***
